# Supplementary material for: Quality of care offered by health care retail markets for medication abortion self-management: Findings from states in Nigeria and India
Source: PLOS Glob Public Health. 2025 Jan 6;5(1):e0003971. doi: 10.1371/journal.pgph.0003971 (PMC11703032; doi:10.1371/journal.pgph.0003971)
Supplement: S1 Table — (DOCX) [file pgph.0003971.s001.docx]

| S1 Table. Description of simulated client profiles |
| --- |

| Nigerian states | **Male simulated client**  A man in his late twenties/early thirties comes to the pharmacy/chemist shop with a problem. His girlfriend is pregnant, and he wants something so that she can end the pregnancy. If asked, his girlfriend conducted a pregnancy test the previous day which came back positive and she hasn’t gotten her period in about 6 weeks. |
| --- | --- |
|  | **Female simulated client**  An unmarried woman in her mid to late 20s comes to the pharmacy/chemist shop with a problem. She is pregnant and wants something to end the pregnancy. If asked, she did a pregnancy test the previous day which was positive and hasn’t gotten her period in about 6 weeks. |
| Indian state | **Male simulated client**  A man in his early thirties comes to the pharmacy/chemist shop with a problem. His wife is pregnant, and he wants something so that she can end the pregnancy. If asked, his wife conducted a pregnancy test the previous day which came back positive and she hasn’t gotten her period in about 6 weeks. They have two children. |
|  | **Female simulated client**  A woman in her late twenties comes to the pharmacy/chemist shop with a problem. Her sister-in-law is pregnant, and she wants something so that she can end the pregnancy. If asked, her sister-in-law conducted a pregnancy test the previous day which came back positive and she hasn’t gotten her period in about 6 weeks. Her sister-in-law and her husband have two children. |
