# Supplementary material for: Quality of care offered by health care retail markets for medication abortion self-management: Findings from states in Nigeria and India
Source: PLOS Glob Public Health. 2025 Jan 6;5(1):e0003971. doi: 10.1371/journal.pgph.0003971 (PMC11703032; doi:10.1371/journal.pgph.0003971)
Supplement: S2 Table — (DOCX) [file pgph.0003971.s002.docx]

S2 Table. Proportion of women aged 15-49 residing in communities with medication abortion pills available in the local retail market in three Nigerian states and an Indian state

| Characteristic | Nigerian states | | Indian state | |
| --- | --- | --- | --- | --- |
|  | N | % | N | % |
| Reside in a community where MA pills are available | 399 | 52.8 | 1000 | 56.8 |
| Reside in a community where MA pills are unavailable | 357 | 47.2 | 762 | 43.2 |
| Total number of reproductive aged women | 756 | 100.0 | 1762 | 100.0 |
