# Supplementary material for: Quality of care offered by health care retail markets for medication abortion self-management: Findings from states in Nigeria and India
Source: PLOS Glob Public Health. 2025 Jan 6;5(1):e0003971. doi: 10.1371/journal.pgph.0003971 (PMC11703032; doi:10.1371/journal.pgph.0003971)
Supplement: S3 Table — (DOCX) [file pgph.0003971.s003.docx]

S3 Table: Dispensing of medication abortion pills by service delivery points (SDPs) disaggregated by gender of the simulated client in three Nigerian states and an Indian state

|  |  | **Nigerian states** | | | | | | **Indian state** | | | | | |
| --- | --- | --- | --- | --- | --- | --- | --- | --- | --- | --- | --- | --- | --- |
| Characteristic | | Facilities offering MA drugs | | Facilities not offering MA drugs | | All facilities | | Facilities offering MA drugs | | Facilities not offering MA drugs | | All facilities | |
| Simulated client gender | | N | % | N | % | N | % | N | % | N | % | N | % |
|  | Female | 39 | 42.4% | 53 | 57.6% | 92 | 100.0 | 14 | 22.2% | 49 | 77.8% | 63 | 100.0 |
|  | Male | 25 | 27.2% | 67 | 72.8% | 92 | 100.0 | 27 | 42.2% | 37 | 57.8% | 64 | 100.0 |
