# Supplementary material for: Quality of care offered by health care retail markets for medication abortion self-management: Findings from states in Nigeria and India
Source: PLOS Glob Public Health. 2025 Jan 6;5(1):e0003971. doi: 10.1371/journal.pgph.0003971 (PMC11703032; doi:10.1371/journal.pgph.0003971)
Supplement: S4 Table — (DOCX) [file pgph.0003971.s004.docx]

S2 Table. Sensitivity Analysis of change in percentage distribution of quality of care indicators if using a more conservative approach (v2) to indicator calculation in Nigeria

| Quality of Care Domains and Indicators | | %-v1 | %-v2 | Relative  difference | Absolute  difference |
| --- | --- | --- | --- | --- | --- |
| **Technical competency** | |  |  |  |  |
|  | Pregnancy confirmation enquiry | 61.7 | 53.2 | 13.8 | 8.5 |
|  | Gestational age enquiry | 70.2 | 51.1 | 27.3 | 19.1 |
|  | **Provided (prompted or unprompted) correct information on:** |  |  |  |  |
|  | Route of drug administration | 25.5 | 8.5 | 66.7 | 17.0 |
|  | Order of pills^1^ | 46.7 | 40.0 | 14.3 | 6.7 |
|  | Interval between taking mifepristone and misoprostol^1^ | 26.7 | 20.0 | 25.0 | 6.7 |
|  | **Discussed (whether prompted or not) the following symptoms:** |  |  |  |  |
|  | Bleeding | 76.6 | 55.3 | 27.8 | 21.3 |
|  | Abdominal cramps | 27.7 | 12.8 | 53.8 | 14.9 |
|  | **Discussed (whether prompted or not) the following side-effects:** |  |  |  |  |
|  | Nausea | 10.6 | 8.5 | 20.0 | 2.1 |
|  | Vomiting | 12.8 | 8.5 | 33.3 | 4.3 |
|  | Diarrhea | 0.0 | 0.0 | -- | 0.0 |
|  | Chills | 0.0 | 0.0 | -- | 0.0 |
|  | Fever | 12.8 | 2.1 | 83.3 | 10.6 |
|  | **Discussed (whether prompted or not) the following warning signs:** |  |  |  |  |
|  | Excessive bleeding | 31.9 | 17.0 | 46.7 | 14.9 |
|  | Bleeding that stops followed by heavy onset | 8.5 | 0.0 | 100.0 | 8.5 |
|  | Little or no bleeding | 0.0 | 0.0 | -- | 0.0 |
|  | Discussed (whether prompted or not) appropriate locations for follow-up care | 48.9 | 25.5 | 47.8 | 23.4 |
| **Information given to client** | |  |  |  |  |
|  | **Provided unprompted correct information on:** |  |  |  |  |
|  | Route of drug administration | 14.9 | 2.1 | 85.7 | 12.8 |
|  | Order of pills | 20.0 | 20.0 | 0.0 | 0.0 |
|  | Interval between mifepristone and misoprostol | 6.7 | 6.7 | 0.0 | 0.0 |
|  | **Discussed unprompted the following symptoms:** |  |  |  |  |
|  | Bleeding | 21.3 | 12.8 | 40.0 | 8.5 |
|  | Abdominal cramps | 4.3 | 2.1 | 50.0 | 2.1 |
|  | **Discussed unprompted the following side-effects:** |  |  |  |  |
|  | Nausea | 4.3 | 4.3 | 0.0 | 0.0 |
|  | Vomiting | 4.3 | 2.1 | 50.0 | 2.1 |
|  | Diarrhea | 0.0 | 0.0 | -- | 0.0 |
|  | Chills | 0.0 | 0.0 | -- | 0.0 |
|  | Fever | 8.5 | 2.1 | 75.0 | 6.4 |
|  | **Discussed unprompted the following warning signs:** |  |  |  |  |
|  | Excessive bleeding | 2.1 | 0.0 | 100.0 | 2.1 |
|  | Bleeding that stops followed by heavy onset | 4.3 | 0.0 | 100.0 | 4.3 |
|  | Little or no bleeding | 0.0 | 0.0 | -- | 0.0 |
|  | Discussed unprompted appropriate locations for follow-up care | 6.4 | 2.1 | 66.7 | 4.3 |
|  | Discussed post-abortion contraception | 12.8 | 6.4 | 50.0 | 6.4 |
| **Client experience** | |  |  |  |  |
|  | Treated respectfully | 89.4 | 85.1 | 4.8 | 4.3 |
|  | Provider indicated verbal disapproval of request | 6.4 | 8.5 | -33.3 | -2.1 |
|  | Provider indicated disapproval of request via negative body language | 8.5 | 10.6 | -25.0 | -2.1 |
| v1: Point assigned if information provided in at least one of the two simulated client interactions at a given facility  v2: point assigned if information provided in both interactions at a given facility | | | | | |
