# Supplementary material for: Quality of care offered by health care retail markets for medication abortion self-management: Findings from states in Nigeria and India
Source: PLOS Glob Public Health. 2025 Jan 6;5(1):e0003971. doi: 10.1371/journal.pgph.0003971 (PMC11703032; doi:10.1371/journal.pgph.0003971)
Supplement: S1 Checklist — (DOCX) [file pgph.0003971.s005.docx]

Inclusivity in global research

PLOS’ policy on inclusivity in global research aims to improve transparency in the reporting of research performed outside of researchers’ own country or community and ensures that PLOS publications reporting global research adhere to high standards for research ethics and authorship. Authors of relevant research articles may be asked to complete the questionnaire below, which outlines ethical, cultural, and scientific considerations specific to inclusivity in global research. This questionnaire may be requested when researchers have travelled to a different country to conduct research, if research uses samples collected in another country, research with Indigenous populations or their lands, or if research is on cultural artefacts. Researchers travelling to another country solely to use laboratory equipment will not normally be required to complete the questionnaire. However, the questionnaire can be requested at the journal’s discretion for any submission – if you have been requested to complete this questionnaire by the PLOS journal you submitted to, please do so.

Please complete the questionnaire below and include this as a Supporting Information file with your manuscript. Note that if your paper is accepted for publication, this checklist will be published with your article in the supporting information files. Please ensure that you reference the checklist in the main body of your manuscript. We suggest adding a subsection ‘Inclusivity in global research’ to your Methods section and adding the following sentence: “Additional information regarding the ethical, cultural, and scientific considerations specific to inclusivity in global research is included in the Supporting Information (SX Checklist)”

The questions have been designed to be applicable to a wide range of study types, and there are subsections for both human subjects research and non-human subjects research. If any of the questions are not relevant to your research please mark them as “N/A” as appropriate.

**Ethical considerations, permits and authorship**

*This section is applicable to all research types.*

Provide details as to who granted permissions and/or consent for the study to take place in the Methods section of your manuscript. This should include the names of **all** ethics boards, governmental organizations, community leaders or other bodies that provided approval for the study. If individuals provided approval refer to these people by their role or title but do not list their name(s).

Reported on page number: Details of all ethical approvals included in section titled “Ethics approvals” on pages 9-10.

If there were any deviations from the study protocol after approval was obtained please provide details of these changes in the Methods section of your manuscript.
Did this study involve local collaborators that are residents of the country where the research was conducted or members of the community studied? If you do not have any authors from said communities, please provide an explanation for this below.

Reported on page number: N/A

Yes, among the seven manuscript authors, two are residents and/or Nigerian nationals, and three are residents and/or Indian nationals.

Everyone listed as an author should meet PLOS’ criteria for authorship and all individuals who meet these criteria should be included in the author byline, rather than the acknowledgements. For further information please see the journal’s Authorship Policy.

**Human subjects research (e.g. health research, medical research, cross-cultural psychology)**

Did you obtain written informed consent from a representative of the local community or region before the research took place? How did you establish who speaks for the community? Details of written informed consent obtained from study participants should be reported separately in the Methods section of your manuscript.

We received permission to conduct the female surveys with reproductive aged women, the service delivery point surveys, and the simulated client study from the National Health Research Ethics Committee of Nigeria, and the Institutional Ethics Committee at our collaborating institution in the Indian state. Approvals were also obtained for the simulated client study from governmental ministries in the three Nigerian states where this study component was implemented.

All female survey respondents provided informed consent (Page 7). For the simulated client study, pharmacy and chemist staff were blinded to study participation to ensure that the interaction captured real experiences that an actual client was likely to have (Page 9).

How did members of the local community provide input on the aims of the research investigation, its methodology, and its anticipated outcome(s)?

The study was designed jointly among the research team members at the Centre for Research Evaluation Resources and Development (Nigeria), the Department of Community Medicine, College of Medicine, University of Ibadan, Ibadan, Oyo (Nigeria), the Indian Institute of Health Management Research, Jaipur (India), and the Department of Population, Family and Reproductive Health, Johns Hopkins Bloomberg School of Public Health, Baltimore (USA).

The simulated client study was conceived in the context of a multi-country abortion project amidst discussions about the important but understudied role of providers in the health care retail market in facilitating access to medication abortion pills to women in India and Nigeria. Our colleagues in Nigeria and India recommended the simulated client approach as a way to capture ground realities, and all co-authors and research staff in Nigeria and India worked collaboratively to design and implement the work, paying particular attention to the unique ethical implications of study activities in each context.

When engaging with the local community, how did you ensure that the informed consent documents and other materials could be understood by local stakeholders?

All study forms were designed, translated into several local languages, tested and refined through multiple rounds of discussions with field teams administering the study activities in each location, and during trainings and piloting phases prior to study implementation.

Will the findings of the research be made available in an understandable format to stakeholders in the community where the study was conducted (e.g. via a presentation, summary report, copies of publications, etc.)? Please provide details of how this will be achieved.

A one-page summary brief containing relevant key findings has previously been shared with all pharmacies and chemist shops eligible to participate in this study in the Nigerian setting. A similar approach was not undertaken in the Indian setting due to concerns among our Indian colleagues that distributing information regarding informal dispensing of medication abortion drugs could induce fear among pharmacy and chemist shop providers, and discourage many of them from stocking the medications. This decision was taken in close consultation with members of the research and practitioner communities in India and the relevant IRB committees overseeing this project.

**Non-human subjects research using specimens/ animals collected as part of the study, or those housed in archival collections. Examples include archaeology, paleontology, botany and zoology.**

Did the permission you obtained from a local authority to perform the study include an agreement on access to outputs and benefit sharing? This may include procedures to enable fair distribution of the benefits and resources arising from the research performed. Please include any details of Prior Informed Consent and Benefit Sharing Agreements obtained. These may be required by field-specific regulations, for example the Convention on Biological Diversity (CBD) and the associated Nagoya Protocol.

N/A

If the material used in your study was imported, please A) provide the year it was imported and B) indicate whether permits were obtained to import/export the materials used, C) provide details of any permits obtained. If this information is not available, please indicate this.

N/A

If you used archival specimens, please state how the material used in your study was acquired by the institute it is held in and provide details of any permits obtained for the original excavations/ sample collection. If this information is not available, please indicate this.

N/A

How was the potential cultural significance of the materials collected in your study to local communities considered in your research design? Were Indigenous peoples and/or local researchers and institutions involved with archaeological excavations / collection of specimens? If so, please provide a description of their involvement.

N/A

If your manuscript includes photographs of human remains please indicate whether authors obtained permission from descendants or affiliated cultural communities to do so.

N/A
