# Supplementary material for: Quality of care offered by health care retail markets for medication abortion self-management: Findings from states in Nigeria and India
Source: PLOS Glob Public Health. 2025 Jan 6;5(1):e0003971. doi: 10.1371/journal.pgph.0003971 (PMC11703032; doi:10.1371/journal.pgph.0003971)
Supplement: Attachment — Submitted filename: Response To Reviewers.docx [file pgph.0003971.s006.docx]

9 November 2023

Dear PLOS GPH Editorial Team,

We are grateful to the two reviewers for their feedback on our manuscript entitled “Quality of care offered by health care retail markets for medical abortion self-management: findings from states in Nigeria and India” (Manuscript ID PGPH-D-23-00768).

Please find on Page 2 a table (Table 1) with each of the reviewers’ and editor’s comments, our responses, and, if applicable, the page and paragraph number(s) where associated changes have been made in the revised manuscript.

Sincerely,

Mridula Shankar (and on behalf of the manuscript’s co-authors)

**Table 1: Reviewer comments and responses**

**Reviewer 1:**

My reviews are attached on the manuscript as comments.

|  | Comments to the author | Authors’ response | Page & paragraph numbers in the updated manuscript |
| --- | --- | --- | --- |
| 1 | Please indicate the proof of ethical approval and ethical considerations | We apologize for not providing additional details regarding the various ethical approvals received for the study. We have now added a separate section titled “Ethics approvals” in the manuscript which provides details related to all the prospective ethics approvals that the study received prior to implementation. | Page 9, lines 233-250. |
| 2 | You talked of face-to-face interviews in your methodology, however, you did not indicate anywhere the results of this qualitative data | We discuss the use of 3 data sources in the manuscript’s methodology section:  (1) The primary data source for this analysis is a simulated client study with pharmacies and chemist shops.  The analysis uses data from two additional sources:  (2) survey data from the same pharmacies and chemist shops (enabling us to compare availability of medication abortion drugs using self-reported survey data and simulated client visit data), and  (3) face-to-face survey data from reproductive aged women (15-49 years) residing in the same geographical areas where the sampled pharmacies and chemist shops were located. This data source enabled us to examine any systematic differences in medication abortion drug availability (using data from the simulated client study) by characteristics of women living in the sampled areas (using survey data from reproductive-aged women).  The paper does not report on qualitative interviews. | Page 6, lines 158-173 |
| 3 | Introduction, Page 3, lines 71-72:  Reviewer comment: Consider revising the language to past tense. | Thank you for this suggestion. We have reviewed the sentence and would like to retain the statement in the present tense as it describes a conceptual and practical nuance of the meanings of medication abortion self-management that remains true in the present time. | Page 3, Lines 76-78 |
| 4 | Introduction, Page 3, lines 75-78:  Reviewer comment: This is confusing, I probably thought you were comparing MA pills not with herbal. Please clarify, I thought it would not add so much to your study | We have revised this sentence to read as follows: “Unguided self-management with MA pills has significantly reduced the health risks previously associated with clandestine abortions outside clinical settings, as evidenced by decreased severe abortion-related morbidity and mortality. | Page 3, Lines 79-81 |
| 5 | Introduction, Page 5, lines 120-124:  Reviewer comment: Please make it continuous statement without (a) and (2) | We have updated our presentation of the aims of the manuscript as follows:  This study has two aims: first, to define, measure and aggregate process indicators of quality that measure technical competency and client experience associated with informal dispensing of MA pills, and second, to assess disparities in the availability of MA pills in the informal sector by socio-demographic characteristics of reproductive-aged women living in communities where these sampled drug vendors are located. | Page 5, lines 129-133 |
| 6 | Discussion, Page 24, lines 484-490:  Reviewer comment: This would best be modified to fit in the recommendation | We have modified the introductory sentence to this paragraph as follows:  “Our study findings have several additional policy, programmatic and research implications.”  We hope that this re-structure will signal to the readers that the paragraph discusses implications/recommendations arising from the study findings. | Page 25, Line 524 |
| 7 | Discussion (Limitations), Page 25, lines 519-521: Reviewer comment: “A good one but I would suggest you think of making suggestion for further studies” | Thank you for making this suggestion. The limitation of this analysis that it does not account for women living on the periphery of an EA and having geographical access to a facility in a neighboring EA, or one close to a workplace, is a difficult limitation to overcome. Unfortunately, it would not be feasible to geocode all the possible pharmacy/chemist access points that women are likely to visit outside of their residential areas. Additionally, given that our analysis indicated considerable under-reporting of stocking of medication abortion drugs, determining whether all the possible access points stock medication abortion drugs would also be a challenging endeavor. Given these practical difficulties, we are unable to provide a suitable alternative approach to overcome this limitation. | N/A |
| 8 | Discussion, Pages 25-26:  Reviewer comment: I would suggest you create different headings for strengths and weaknesses | Thank you for this suggestion. We have now created a separate sub-heading titled “Study Limitations and Strengths” | Page 26, Line 549 |
| 9 | Conclusion, Page 26:  Reviewer comment: I would suggest you start by highlighting the main object which is quality of care in the first paragraph to give an insight to readers as your main findings are defined. Also make the paragraph more summarised | Thank you for this suggestion. We have now modified the conclusion to bring forward our findings regarding the quality of care associated with informal dispensing of medication abortion pills.  In line with the Reviewer’s suggestion to make this paragraph more concise, we have deleted information regarding the different legal contexts of India and Nigeria as this information is secondary to our main take-away that there needs to be changes in the structural and healthcare environments to better integrate trained pharmacy provision of MA pills to facilitate the most favorable health outcomes. | Page 27, Lines 576-589 |

**Reviewer 2**

I have reviewed the research article "Quality of care offered by health care retail markets for medical abortion self-management: findings from states in Nigeria and India". The authors present new, interesting and policy-relevant data and analysis on the state of MA provision quality in two different contexts where self-managed medical abortion is common. The methodology is clearly presented and appropriate, with data provided supporting claims that provision is high, but quality is variable, and when totally unprompted, pretty low. Underlying data was not provided, understandably, do to safety/privacy concerns, which was stipulated. The paper will provide useful evidence to shape regulatory frameworks, particularly as data on the quality of MA provision from retail outlets is limited. The paper is well written and easy to follow, but there are two very minor copy edit comments:

|  | Comments to the author | Authors’ response | Page & paragraph numbers in the updated manuscript |
| --- | --- | --- | --- |
| 1 | There are (it seems) formula errors in the table on page 17 (i.e. "REF"); | REF stands for “Reference Category”; we apologize for not noting this in the table legend previously. We have now indicated the full form of the REF acronym in the table legend. | Page 18, Legend of Table 3 |
| 2 | There is a typo/extra word on page 3 at the bottom "Yet, research in Asian, Latin American and sub-Saharan African countries suggests indicates information provided during...". | Thank you for bringing this typo to your notice. We have now corrected it by deleting the word “indicates”. | Page 3, line 85 |

**Journal Requirements**

|  | Comments to the author | Authors’ response | Page & paragraph numbers in the updated manuscript |
| --- | --- | --- | --- |
| 1 | Please review your reference list to ensure that it is complete and correct. If you have cited papers that have been retracted, please include the rationale for doing so in the manuscript text, or remove these references and replace them with relevant current references. Any changes to the reference list should be mentioned in the rebuttal letter that accompanies your revised manuscript. If you need to cite a retracted article, indicate the article’s retracted status in the References list and also include a citation and full reference for the retraction notice. | Thank you. The reference list has been checked. One new reference (citation below) has been added to the following sentence in the Introduction – “Yet, research in Asian, Latin American and sub-Saharan African countries suggests that information provided during pill purchases is often inadequate or incorrect…”  Reference added (Ref # 11):  Otsin MNA, Black K, Hooker L, Taft AJ. Pharmacy dispensing of abortion pills in Ghana: experiences of pharmacy workers and users. BMJ Sexual & Reproductive Health. 2023.  The new reference has been added as this is a newly published manuscript with data from Ghana that provides additional empirical support for the statement above. | Page 3, Line 85, reference #11 |
| 2 | Please include a complete copy of PLOS’ questionnaire on inclusivity in global research in your revised manuscript. | Completed questionnaire included as Supporting Information file (S1 Checklist) with manuscript. |  |
| 3 | We noticed that you used "data not shown" in the manuscript. We do not allow these references, as the PLOS data access policy requires that all data be either published with the manuscript or made available in a publicly accessible database. Please amend the supplementary material to include the referenced data or remove the references. | We have now included the data referenced in the text within 2 Supplementary Tables (S2 Table and S3 Table) included as part of the Supporting Information |  |

**Additional Changes**

|  | Changes | Page & paragraph numbers in the updated manuscript |
| --- | --- | --- |
| 1 | Updated current address of corresponding author | Page 1, Line 28 |
| 2 | Replaced “medical abortion” with “medication abortion” throughout the manuscript. We have instituted this change in alignment with 2023 guidance on standardization of abortion nomenclature and the associated recommendation to use the term *medication abortion* when referring to the use of medications including mifepristone and/or misoprostol to induce an abortion. The citation for this guidance is included below:  Upadhyay UD, Coplon L, Atrio JM. Society of Family Planning Committee Statement: Abortion nomenclature. Contraception. 2023 Oct 1;126. | Throughout manuscript |
