# Supplementary material for: Quality of care offered by health care retail markets for medication abortion self-management: Findings from states in Nigeria and India
Source: PLOS Glob Public Health. 2025 Jan 6;5(1):e0003971. doi: 10.1371/journal.pgph.0003971 (PMC11703032; doi:10.1371/journal.pgph.0003971)
Supplement: Attachment — Submitted filename: Response To Reviewers_R2.1.docx [file pgph.0003971.s008.docx]

7 May 2024

Dear PLOS GPH Editorial Team,

We are grateful to the reviewer who has provided a second round of feedback on our manuscript titled “Quality of care offered by health care retail markets for medication abortion self-management: findings from states in Nigeria and India” (Manuscript ID PGPH-D-23-00768R1).

Please find from pages 2-26 our responses to each of the reviewer’s and editor’s comments, and, if applicable, the page and paragraph number(s) where associated changes have been made in the revised manuscript.

Thank you for your time and consideration to our manuscript.

Sincerely,

Mridula Shankar (and on behalf of the manuscript’s co-authors)

**Table 1: Reviewer comments and responses**

**Reviewer 1:**

|  | Reviewer comments to the authors | Authors’ response | Page & paragraph numbers in the updated manuscript |
| --- | --- | --- | --- |
| 1 | Abstract  The abstract is good. However, it needs sectioning into sub-headings for easy reading | Thank you for this suggestion. We have retained the abstract in its current form to abide by the journal’s instructions on the journal’s webpage titled “Submission Guidelines”, available here: https://journals.plos.org/globalpublichealth/s/submission-guidelines#loc-manuscript-organization  We have included the applicable instructions below:  While the Abstract is conceptually divided into three sections (Background, Methodology/Principal Findings, and Conclusions/Significance), do not apply these distinct headings to the Abstract within the article file. | N/A |
| 2 | There is need for authors to keep the manuscript as concise as possible to enable readers to capture relevant details with ease. | Thank you for this feedback. We have gone through the manuscript several times and made some minor edits to reduce word count. We feel that any further content reduction will negatively impact manuscript quality. Since we use a combination of data sources, unique sampling strategies, and operationalize, measure and report on process indicators of quality in two geographically distinct settings, this manuscript is of a somewhat longer length than usual. |  |
| 3 | Comment in manuscript:  It would be great to just state the aims of the study and connect with “and” | We have modified the final paragraph of the Introduction in line with this suggestion. It now reads as follows:  This study as two aims: to define, measure and aggregate process indicators of quality that measure technical competency and client experience associated with informal dispensing of MA pills, and, to assess disparities in the availability of MA pills in the informal sector by socio-demographic characteristics of reproductive-aged women living in communities where these sampled drug vendors are located. | Page 5, Lines 76-78 |
| 4 | Methods Section  Well-revised | Thank you | N/A |
| 5 | Results section  Well summarized and answered relevant questions | Thank you | N/A |
| 6 | Discussion section  More clearer, however, the authors needed to bring out the implications and possible recommendations in each finding | Thank you for this feedback. We have reviewed the Discussion section and made some minor edits so that the implications and/or recommendations from our findings are more clearly articulated. However, we have not made major content changes as the co-author team felt that the implications and recommendations associated with this study are already presented in this section. We have highlighted the various instances in the Discussion where we have drawn out the implications and recommendations from this work.   1. In relation to our finding of lower than anticipated dispensing of medication abortion drugs in the Indian state, compared to national estimates, we note the current regulatory context of the Indian state and the implications on dispensing practices as follows (Pg 22, lines 447-452):   *With a history of gender-biased sex ratios in certain Indian states, the government’s prioritizing of law enforcement against fetal sex determination has overshadowed the implementation of services in line with the legal framework on abortion. Such measures have given rise to both uncertainty about legal grounds for abortion and created a “chilling effect” on providers, including drug retailers, who fearing prosecution, may increasingly stop stocking the combination regimen.*   2. Associated with our finding that some pharmacies and chemist shops in the Indian state were providing ayurvedic pill preparations in addition to or instead of recommended medication abortion regimens, we note the following implication (Pg 23, lines 464-468):   *To the extent that these dispensing practices are shaped by the “tug” of punitive legal and policy environments in which providers operate while responding to the “pull” of social and financial incentives to meet ongoing demand, women in these settings are at risk of preventable abortion-related morbidity when non-recommended products are dispensed in substitution for MA pills.*   3. As an implication of our findings regarding variation in the distribution of medication abortion drug availability by characteristics of reproductive-aged women, we note the following implication (Page 23, lines 472-480)   *These findings suggest the persistent roles that structural factors play in shaping inequities in access to recommended abortion pills, even in the informal health care environment, and consequently in the risks and harms experienced across the spectrum of self-managed abortions. Such inequities may also inform method selection, with drug vendors deciding which pills to stock depending on the size and location of their outlets (30). Even with the introduction of mifepristone into the retail market in Nigeria, the substantial price difference between the misoprostol-only and combined regimens will likely influence decisions among retailers and clients alike on the type of pills stocked or requested for self-managed termination.*   4. In relation to our finding regarding the variations in costs of pills for self-management of medication abortion, we highlight the following implication (Page 24, 489-493):   *Self-management of abortion in both these contexts relies entirely on self-financing as abortion remains a contested health service. Our findings suggest that in the absence of integrating modes of supply-side financing, existing inequities in the risk of unsafe abortion driven by dispensing practices that reduce out-of-pocket costs but involve the sale of ineffective or harmful products will likely persist.*   5. Our finding regarding statistically significant differences in dispensing behaviors by client gender motived the following recommendation that we make (Page 24, lines 499-501):   *We recommend that future research investigate the drivers of differential dispensing practices in various contexts to better illuminate the role of gender and its intersection with other structural factors in influencing dispensing behaviors.*   6. Our final paragraph in the Discussion, reproduced below, is dedicated to the broader policy, programmatic and research implications of this work (Pages 25-26, lines 522-544)   *Our study findings have several additional policy, programmatic and research implications. The WHO’s normative guidance on self-care interventions for sexual and reproductive health and their updated 2022 abortion care guidelines have clearly articulated a role for self-management of MA as a recommended service delivery strategy. Both in Nigeria and India, current legal frameworks governing delivery of abortion care criminalize self-management of abortion, making it impossible for health systems in either country to develop and implement policies that align with current public health guidance on abortion self-care. Our study reiterates the critical and growing role of pharmacy and chemist staff in facilitating access to misoprostol and mifepristone. If they continue to operate outside regulatory boundaries, evidence consistently suggests that technical quality of accompanying care will remain sub-standard. What works to improve dispensing behaviors is a question that remains to be answered as evaluations of training programs have demonstrated improvements in knowledge, but translation to behavior change has been mixed (43-45). To take a patient-centered approach to abortion self-management, health systems in both countries will need to pivot away from clinical “provider-to-receiver” models of care to health promotion approaches that prioritize improving user knowledge and education. Efforts directed at raising the knowledge of users themselves, for instance with label inserts, are still in the nascent stages of development but appear to have potential (46). Finally, accompaniment models of self-managed MA, where individuals receive evidence-based information from trained counsellors have been successful not only in bridging some critical gaps in abortion care access but also demonstrating high levels of safety and effectiveness (4, 47, 48). Research is needed to generate data on the current reach of these services, and accompanying economic evaluations can help inform the allocation of financial and human resources to take successful interventions to scale.* | Page 22, lines 447-452  Page 23, lines 464-468  Page 23, lines 472-480  Page 24, 489-493  Page 24, lines 499-501  Pages 25-26, lines 522-544 |
| 7 | There was a need to further show evidence of ethical approval as renewed during the study time. | In the table below, we have provided a chronological overview of all the ethics approvals and data collection dates for each stage and country, and have included as appendices, the respective ethics approvals for each study component. We are a little unclear about this reviewer comment regarding renewal of ethics approval, noting that all participant data were collected prospectively after ethics approvals were received and additionally that all participant data were collected after the start date of ethics approval and before the end date of ethics approval, if an end-date is stated in the ethics approval letter. Once collected, the data were immediately de-identified, and all data analyses were conducted using de-identified data, in accordance with ethics approvals. | N/A |

| # | Study component | Study location | Ethics approval date | Ethics committee | Data collection dates | Ethics approval document |
| --- | --- | --- | --- | --- | --- | --- |
| 1 | The Performance Monitoring and Accountability 2020 (PMA 2020) national female and SDP surveys | Nigeria | 2 January 2018 | National Health Research Ethics Committee of Nigeria | April – May 2018 | Appendix 1.1 |
| 2 | The Performance Monitoring and Accountability 2020 (PMA 2020) female and SDP surveys | Rajasthan, India | 16 February 2018 | Institutional Review Board for Protection of Human Subjects, Indian Institute of Health Management Research University | April – June 2018 | Appendix 1.2 |
| 3 | The Performance Monitoring and Accountability 2020 (PMA 2020) female surveys  ***Note:*** Johns Hopkins IRB deemed the SDP surveys as non-human subjects research since facility data collected on staffing, stock of medications, services provided etc., are unrelated to personal information, perspectives, or opinions. | Nigeria and Rajasthan, India | 27 March 2018 | Johns Hopkins Bloomberg School of Public Health | April – June 2018 | Appendix 1.3 |
| I | PMA 2020 simulated client study | Study sites in Nigeria (Anambra, Kaduna and Rivers states) and India (Rajasthan) | 26 June 2018 & 11 July 2018 | Johns Hopkins Bloomberg School of Public Health | Please see state-based data collection dates below | Appendix 2.1A  Appendix 2.1B |
| 3 | PMA 2020 simulated client study | Anambra state, Nigeria | 4 June 2018 | Ministry of Health, Anambra state | July 2018 | Appendix 2.2 |
| 4 | PMA 2020 simulated client study | Kaduna state, Nigeria | 13 July 2018 | Ministry of Health and Human Services, Kaduna state | August 2018 | Appendix 2.3 |
| 5 | PMA 2020 simulated client study | Rivers state, Nigeria | 10 July 2018 | Government of Rivers state of Nigeria | Mid-July-August 2018 | Appendix 2.4 |
| 6 | PMA 2020 simulated client study | Rajasthan, India | 30 June 2018 | Institutional Review Board for Protection of Human Subjects, Indian Institute of Health Management Research University | Mid-July – August 2018 | Appendix 2.5 |

Appendix 1.1

Appendix 1.2

Appendix 1.3

Appendix 2.1A

Appendix 2.1B

Appendix 2.2

Appendix 2.3

Appendix 2.4

Appendix 2.5

**Journal Requirements**

|  | Comments to the author | Authors’ response | Page & paragraph numbers in the updated manuscript |
| --- | --- | --- | --- |
| 1 | Please review your reference list to ensure that it is complete and correct. If you have cited papers that have been retracted, please include the rationale for doing so in the manuscript text, or remove these references and replace them with relevant current references. Any changes to the reference list should be mentioned in the rebuttal letter that accompanies your revised manuscript. If you need to cite a retracted article, indicate the article’s retracted status in the References list and also include a citation and full reference for the retraction notice. | Thank you. The reference list has been checked and no changes have been made. |  |
| 2 | Please include a complete copy of PLOS’ questionnaire on inclusivity in global research in your revised manuscript. | The completed questionnaire is included as a Supporting Information file (S1 Checklist) and referenced in the manuscript. | Page 10, line 248 |
| 3 | Please amend your detailed Financial Disclosure statement. This is published with the article. It must therefore be completed in full sentences and contain the exact wording you wish to be published.  a. State the initials, alongside each funding source, of each author to receive each grant. b. If any authors received a salary from any of your funders, please state which authors and which funders.  If you did not receive any funding for this study, please simply state: “The authors received no specific funding for this work. | The Funding Disclosure statement is as follows:  This study was funded by an Anonymous Donor (grant number 127941) and the Bill & Melinda Gates Foundation (grant number OPP10709004, PI not a co-author) via grants to Performance Monitoring for Action (https://www.pmadata.org) at the Johns Hopkins Bloomberg School of Public Health. The funders had no role in study design, data collection and analysis, decision to publish, or preparation of the manuscript. |  |
