# Supplementary material for: Quality of care offered by health care retail markets for medication abortion self-management: Findings from states in Nigeria and India
Source: PLOS Glob Public Health. 2025 Jan 6;5(1):e0003971. doi: 10.1371/journal.pgph.0003971 (PMC11703032; doi:10.1371/journal.pgph.0003971)
Supplement: Attachment — Submitted filename: Response To Reviewers.docx [file pgph.0003971.s010.docx]

28 October 2024

Dear Dr Tucker,

My apologies for the delay in submitting our revised manuscript. We are grateful to the reviewer who has provided another round of feedback on our manuscript titled “Quality of care offered by health care retail markets for medication abortion self-management: findings from states in Nigeria and India” (Manuscript ID PGPH-D-23-00768R3).

We have addressed each comment made by the reviewer in Table 1, and, where applicable, noted the page and paragraph numbers where changes have been made in the revised manuscript. We have addressed Journal Requirements in Table 2 and included additional changes in Table 3.

Thank you for your time and consideration of our manuscript.

Sincerely,

Mridula Shankar (and on behalf of the manuscript’s co-authors)

**Table 1: Reviewer comments and responses**

**Reviewer 1:**

|  | Reviewer comments to the authors | Authors’ response | Page & paragraph numbers in the updated **track changes** manuscript |
| --- | --- | --- | --- |
| 1 | Abstract  Try to section abstract into Background, objectives, methodology, results, conclusion for readers to be clearer | Thank you for this suggestion. We have retained the abstract in its current form to abide by the journal’s instructions on the journal’s webpage titled “Submission Guidelines”, available here: https://journals.plos.org/globalpublichealth/s/submission-guidelines#loc-manuscript-organization  We have included the applicable instructions below:  While the Abstract is conceptually divided into three sections (Background, Methodology/Principal Findings, and Conclusions/Significance), do not apply these distinct headings to the Abstract within the article file. | N/A |
| 2 | The background is well structured, coherent and relevant; however, it does not capture well contextual statistics at global, regional and the national area. It could be improved | Thank you for this feedback. We have included some statistical data on the frequency of abortion and safety estimates in paragraph 1 of the Introduction (highlighted text below):  Yearly, four in 100 reproductive-aged women undergo an abortion (1). In countries where legal provision of abortion is restricted to narrow grounds and/or where health systems are poorly resourced, peoples’ reliance on clandestine abortion has motivated a longstanding public health focus on abortion safety (2). Safety estimates suggest that 45% of abortions globally are not safe, and the risk of unsafe abortions is disproportionately experienced by women in low-and middle-income regions (3).  We have also added abortion rates for Nigeria and India in the section on Study Contexts as highlighted below:  Both abortion laws and the availability of safe abortion services differ in the two study settings. The legal framework in Nigeria only permits abortion if continuing the pregnancy puts a woman’s life at risk. Nevertheless, abortion is common (33 per 1000 reproductive-aged women (29)), clandestine, and associated safety remains poor (30).  In India, the law is more permissive, allowing women to terminate pregnancies for socio-economic reasons in addition to medical risks. The abortion rate is 48 per 1000 reproductive-aged women (29), but abortion service provision within the public sector is limited, particularly in primary care facilities (33, 34). | Page 3, line 57  Page 3, lines 60-62  Page 6, line 130  Page 6, line 142 |
| 3 | The study context is clear. However, the design seem not to be clear. It would be better to articulate the study design employed in the opening stages to tune readers in comparing with other literature as available | Thank you for this feedback. In the “Data Sources and Sampling” section, we noted at the outset that the study uses a simulated client study design. However, as the simulated client study is nested within larger population-based individual and health facility-based surveys, that provide the sampling frame and contribute data to this analysis, the overall design is somewhat complex. We have chosen to retain this section as is, to adequately convey each of the data sources of this analysis for the purposes of transparency. | N/A |
| 4 | Indicate whether these indicators were adopted or developed by the researchers | As mentioned in the section on Quality of care measures, the indictors were developed by the research team using the 2022 World Health Organization abortion care guideline as reference. This detail has now been included in the Table 1 title as follows:  Table 1. Construction of domains and indicators for process measures of quality of care informed by WHO’s 2022 abortion care guideline. | Page 11, lines 252-253 |
| 5 | The results are well presented and clear to connect to the concept under study. There is need to articulate the statistical power utilized in the advanced analysis | Thank you for this feedback. We have now provided the estimates for the adjusted odds ratios and associated 95% confidence intervals for the results of the multivariable adjusted models as highlighted below:  In the multivariable adjusted models, women in Nigeria with secondary (odds ratio (OR): 10.94, 95% confidence interval (CI): 1.86-33.38) or higher education (OR: 17.61, 95% CI: 2.31, 48.05) and women in the second highest (OR: 15.83, 95% CI: 1.49-135.90) and highest wealth quintiles (OR: 15.29, 95% CI: 1.44-140.33) had significantly higher odds of living in an EA with availability of MA pills compared to women who never went to school and the poorest women (Table 3). In the Indian state, having any wealth compared to being in the poorest quintile, remained independently associated with MA pill availability in EAs (second poorest quintile: OR: 2.5, 95% CI: 1.07-5.58; wealthiest quintile: OR: 17.51, 95% CI: 3.64-57.47). | Page 17, lines 337-345 |
| 6 | The discussion is well articulated and the justifications and implications are well provided for. However, the researchers needed to provide more comparative, cultural and ethical-legal aspects of the reasons more especially in the states. | Thank you for this feedback. We have reviewed the Discussion section and highlighted the various instances where we have offered comparative interpretation of our findings within the larger legal, socio-cultural and health-systems contexts.   1. In relation to our finding of lower than anticipated dispensing of medication abortion drugs in the Indian state, compared to national estimates, we note the current regulatory context of the Indian state and role of government intervention to address gender-biased sex ratios and the resulting implications on dispensing practices as follows:   *With a history of gender-biased sex ratios in certain Indian states, the government’s prioritizing of law enforcement against fetal sex determination has overshadowed the implementation of services in line with the legal framework on abortion. Such measures have given rise to both uncertainty about legal grounds for abortion and created a “chilling effect” on providers, including drug retailers, who fearing prosecution, may increasingly stop stocking the combination regimen.*   2. Associated with our finding that some pharmacies and chemist shops in the Indian state were providing ayurvedic pill preparations in addition to or instead of recommended medication abortion regimens, we note the following implication:   *To the extent that these dispensing practices are shaped by the “tug” of punitive legal and policy environments in which providers operate while responding to the “pull” of social and financial incentives to meet ongoing demand, women in these settings are at risk of preventable abortion-related morbidity when non-recommended products are dispensed in substitution for MA pills.*   3. As an implication of our findings regarding variation in the distribution of medication abortion drug availability by characteristics of reproductive-aged women, we note the following implication:   *These findings suggest the persistent roles that structural factors play in shaping inequities in access to recommended abortion pills, even in the informal health care environment, and consequently in the risks and harms experienced across the spectrum of self-managed abortions. Such inequities may also inform method selection, with drug vendors deciding which pills to stock depending on the size and location of their outlets (30). Even with the introduction of mifepristone into the retail market in Nigeria, the substantial price difference between the misoprostol-only and combined regimens will likely influence decisions among retailers and clients alike on the type of pills stocked or requested for self-managed termination.*   4. In relation to our finding regarding the variations in costs of pills for self-management of medication abortion, we highlight the following implication:   *Self-management of abortion in both these contexts relies entirely on self-financing as abortion remains a contested health service. Our findings suggest that in the absence of integrating modes of supply-side financing, existing inequities in the risk of unsafe abortion driven by dispensing practices that reduce out-of-pocket costs but involve the sale of ineffective or harmful products will likely persist.*   5. Our finding regarding statistically significant differences in dispensing behaviors by client gender motived the following recommendation that we make:   *We recommend that future research investigate the drivers of differential dispensing practices in various contexts to better illuminate the role of gender and its intersection with other structural factors in influencing dispensing behaviors.*   6. Our final paragraph in the Discussion, reproduced below, is dedicated to the broader policy, programmatic and research implications of this work:   *Our study findings have several additional policy, programmatic and research implications. The WHO’s normative guidance on self-care interventions for sexual and reproductive health and their updated 2022 abortion care guidelines have clearly articulated a role for self-management of MA as a recommended service delivery strategy. Both in Nigeria and India, current legal frameworks governing delivery of abortion care criminalize self-management of abortion, making it impossible for health systems in either country to develop and implement policies that align with current public health guidance on abortion self-care. Our study reiterates the critical and growing role of pharmacy and chemist staff in facilitating access to misoprostol and mifepristone. If they continue to operate outside regulatory boundaries, evidence consistently suggests that technical quality of accompanying care will remain sub-standard. What works to improve dispensing behaviors is a question that remains to be answered as evaluations of training programs have demonstrated improvements in knowledge, but translation to behavior change has been mixed (43-45). To take a patient-centered approach to abortion self-management, health systems in both countries will need to pivot away from clinical “provider-to-receiver” models of care to health promotion approaches that prioritize improving user knowledge and education. Efforts directed at raising the knowledge of users themselves, for instance with label inserts, are still in the nascent stages of development but appear to have potential (46). Finally, accompaniment models of self-managed MA, where individuals receive evidence-based information from trained counsellors have been successful not only in bridging some critical gaps in abortion care access but also demonstrating high levels of safety and effectiveness (4, 47, 48). Research is needed to generate data on the current reach of these services, and accompanying economic evaluations can help inform the allocation of financial and human resources to take successful interventions to scale.* | Page 22, lines 442-447  Page 23, lines 453-457  Page 23, lines 461-469  Page 24, 477-481  Page 24, lines 487-489  Pages 25-26, lines 506-527 |

**Table 2: Journal Requirements**

|  | Comments to the author | Authors’ response | Page & paragraph numbers in the updated **track changes** manuscript |
| --- | --- | --- | --- |
| 1 | Please review your reference list to ensure that it is complete and correct. If you have cited papers that have been retracted, please include the rationale for doing so in the manuscript text, or remove these references and replace them with relevant current references. Any changes to the reference list should be mentioned in the rebuttal letter that accompanies your revised manuscript. If you need to cite a retracted article, indicate the article’s retracted status in the References list and also include a citation and full reference for the retraction notice. | Thank you. The reference list has been checked and 7 new references have been added to the manuscript.   1. In response to Reviewer 1’s suggestion to include contextual statistics at the global, regional and national levels, the following highlighted references are included along with the newly added text.   *Yearly, four in 100 reproductive-aged women undergo an abortion* (1)  **Reference #1:** Bearak J, Popinchalk A, Ganatra B, Moller AB, Tunçalp Ö, Beavin C, et al. Unintended pregnancy and abortion by income, region, and the legal status of abortion: estimates from a comprehensive model for 1990-2019. Lancet Glob Health. 2020;8(9):e1152-e61  *Safety estimates suggest that 45% of abortions globally are not safe, and the risk of unsafe abortions is disproportionately experienced by women in low-and middle-income regions* (3)  **Reference #3:** Ganatra B, Gerdts C, Rossier C, Johnson BR, Jr., Tuncalp O, Assifi A, et al. Global, regional, and subregional classification of abortions by safety, 2010-14: estimates from a Bayesian hierarchical model. Lancet. 2017;390(10110):2372-81.  *The legal framework in Nigeria only permits abortion if continuing the pregnancy puts a woman’s life at risk. Nevertheless, abortion is common (33 per 1000 reproductive-aged women* (29)), *clandestine, and associated safety remains poor* (30).  *In India, the law is more permissive, allowing women to terminate pregnancies for socio-economic reasons in addition to medical risks. The abortion rate is 48 per 1000 reproductive-aged women* (29), *yet abortion service provision within the public sector is limited, particularly in primary care facilities* (33, 34).  **Reference #29:** Bearak JM, Popinchalk A, Beavin C, Ganatra B, Moller A-B, Tunçalp Ö, et al. Country-specific estimates of unintended pregnancy and abortion incidence: a global comparative analysis of levels in 2015–2019. BMJ global health. 2022;7(3):e007151.   2. An additional reference (# 16) has been added for a paper published with data from Madagascar that supports the following statement in the introduction:   *Yet, research in Asian, Latin American and sub-Saharan African countries suggests that information provided during pill purchases is often inadequate or incorrect* (10-13), *with dosages provided not meeting technical recommendations* (9, 14-16).  Reference #16: Pourette D, Mattern C, Ratovoson R, Raharimalala P. Complications with use of misoprostol for abortion in Madagascar: between ease of access and lack of information. Contraception. 2018;97(2):116-21   3. The statement in the introduction regarding the WHO’s definition of quality of care was missing the WHO reference (#21). It has now been added as follows:   *With this reality, an expanded focus on quality — which the WHO defines as care that is safe, effective, timely, efficient, equitable and people-centred — can serve as an integrating framework to appraise multiple components of abortion care that are central to its experience and amenable to improvements* (21)  **Reference #21:** World Health Organization. Quality of Care: A Process for Making Strategic Choices in Health Systems. Geneva: Switzerland: World Health Organization; 2006.   4. A new paper on quality of abortion care has been published in the Lancet during the period of this peer review. This reference (#27) has been added to the following sentence in the Introduction:   *Despite growing acknowledgment of the integral role played by the informal health care sector in care provision* (24), *including in the case of abortion* *(25), systematic efforts to widen the scope of quality measurement to include informal providers has only been a recent endeavor* (11, 12, 26, 27)  **Reference #27:** Pearson EE, Chakraborty NM, Baum SE, Menzel JL, Dijkerman S, Chowdhury R, et al. Developing and validating an abortion care quality metric for facility and out-of-facility settings: an observational cohort study in Bangladesh, Ethiopia, and Nigeria. Eclinicalmedicine. 2023;66   5. An additional reference has been added to the statement in the Discussion regarding evidence on the effect of training programs for pharmacy providers on dispensing medication abortion drugs. The reference (#52) has been added to the following sentence:   *What works to improve dispensing behaviors is a question that remains to be answered as evaluations of training programs have demonstrated improvements in knowledge, but translation to behavior change has been mixed* (49-52)  **Reference #52:** Tamang A, Puri M, Masud S, Karki DK, Khadka D, Singh M, et al. Medical abortion can be provided safely and effectively by pharmacy workers trained within a harm reduction framework: Nepal. Contraception. 2018;97(2):137-43 | Page 3, line 57  Page 3, line 62  Page 6, line 130  Page 6, line 143  Page 4, line 83  Page 4, line 94  Page 5, line 111  Page 25, line 517 |
| 2 | Please include a complete copy of PLOS’ questionnaire on inclusivity in global research in your revised manuscript. | The completed questionnaire is included as a Supporting Information file (S1 Checklist) and referenced in the manuscript. | Referenced on page 10, line 238 |

**Table 3: Additional Changes**

|  | Changes | Page & paragraph numbers in the updated **track changes** manuscript |
| --- | --- | --- |
| 1 | Updated current address of corresponding author | Page 1, line 26 |
| 2 | Revised limitations section of the Discussion to highlight that the low precision of the effect estimates from the regression analysis is due to the small sample sizes. | Page 26, lines 544-545 |
